# The persistence and stabilization of auxiliary genes in the human skin virome

**DOI:** 10.1186/s12985-023-02012-3

**Published:** 2023-03-22

**Authors:** Ema H. Graham, Wesley A. Tom, Alison C. Neujahr, Michael S. Adamowicz, Jennifer L. Clarke, Joshua R. Herr, Samodha C. Fernando

**Affiliations:** 1 PhD Program in Complex Biosystems, University of Nebraska, 3940 Fair St, C220K, Lincoln, NE 68583 USA; 2 Department of Animal Science, University of Nebraska, Lincoln, NE 68583 USA; 3 School of Biological Sciences, University of Nebraska, Lincoln, NE 68588 USA; 4 College of Agricultural Sciences and Natural Resources, University of Nebraska, Lincoln, NE 68583 USA; 5 Department of Statistics, University of Nebraska, Lincoln, NE 68588 USA; 6 Food Science and Technology Department, University of Nebraska, Lincoln, NE 68588 USA; 7 Department of Plant Pathology, University of Nebraska, Lincoln, NE 68503 USA; 8 Center for Plant Science Innovation, University of Nebraska, Lincoln, NE 68503 USA; 9 Nebraska Center for Virology, University of Nebraska, Lincoln, NE 68583 USA

**Keywords:** Virome, Metagenome, Human skin, Bacteriophage, Antibiotic resistance

## Abstract

**Background:**

The human skin contains a diverse microbiome that provides protective functions against environmental pathogens. Studies have demonstrated that bacteriophages modulate bacterial community composition and facilitate the transfer of host-specific genes, potentially influencing host cellular functions. However, little is known about the human skin virome and its role in human health. Especially, how viral-host relationships influence skin microbiome structure and function is poorly understood.

**Results:**

Population dynamics and genetic diversity of bacteriophage communities in viral metagenomic data collected from three anatomical skin locations from 60 subjects at five different time points revealed that cutaneous bacteriophage populations are mainly composed of tailed Caudovirales phages that carry auxiliary genes to help improve metabolic remodeling to increase bacterial host fitness through antimicrobial resistance. Sequence variation in the MRSA associated antimicrobial resistance gene, *erm(C)* was evaluated using targeted sequencing to further confirm the presence of antimicrobial resistance genes in the human virome and to demonstrate how functionality of such genes may influence persistence and in turn stabilization of bacterial host and their functions.

**Conclusions:**

This large temporal study of human skin associated viruses indicates that the human skin virome is associated with auxiliary metabolic genes and antimicrobial resistance genes to help increase bacterial host fitness.

**Supplementary Information:**

The online version contains supplementary material available at 10.1186/s12985-023-02012-3.

## Introduction

Viruses are the most abundant biological entity on this planet. It is estimated that the ocean alone contains 100-billion times as many viruses than there are grains of sand on Earth [1]. In addition to being the most abundant biological entity, viruses are arguably the most diverse biological entities on Earth due to their mutation rate and speed of replication. Despite their abundance and diversity, our understanding of viral ecology and function is in its infancy.

Viruses infect all forms of life and may influence host population dynamics, yet many viruses' functional influence has yet to be elucidated. Viruses may influence the host organism by increasing fitness through genetic transfer and expression of host-specific genes that confer fitness [2–7]. Most microbiome studies have focused on the bacteriome [8, 9]. However, the viral component of the microbiome is an essential factor contributing to community assembly and function. It has been demonstrated that bacteriophages can acquire genes that may provide the host with advantageous evolutionary adaptations, such as biofilm inducing genes, proviral genes that promote cell repair, toxin and virulence genes that promote host fitness [4, 6, 10–12]. Additionally, specific bacteriophage genomes retain rate-limiting metabolic genes that increase host fitness [4, 6, 13, 14]. Acquisition and expression of beneficial host metabolic genes were first described in marine bacteriophage populations, where marine bacteriophages were shown to carry auxiliary metabolic genes that contribute to photosynthetic processes [15].

For animals and humans, the skin is the first barrier of defense against external pathogens. Shifts in the population dynamics of the skin microbiome have been shown to contribute to human diseases such as infections caused by *Staphylococcus aureus* or *Streptococcus pyogenes* [8, 16]. Although studies have speculated about complex interactions of the human skin virome and its effects on bacterial pathogen colonization, the magnitude of host-viral interactions and the role of viruses in shaping the skin microbiome is poorly understood [17–20]. Especially in regards to the viral contribution to human disease. Alternatively, bacteriophages have long been of interest in treating bacterial diseases and conditions by controlling bacterial population dysbiosis and antimicrobial resistance [21–24], yet little is known about skin phage diversity and their role in shaping the skin microbiome.

Previous studies have demonstrated that bacteriophages on the skin carry auxiliary metabolic genes (AMGs), which have the potential to bolster host fitness and increase microbiome function [17, 25]. We previously reported the persistence and abundance of viruses, including bacteriophages on the skin virome over time [26]. In this present study, we investigated the diversity of the human skin virome and identified auxiliary metabolic genes present within the skin virome and their effect on microbiome function and host fitness. To this end, we evaluated the skin virome longitudinally across 60 human individuals across three different anatomical locations at five different time points by expanding the dataset developed previously by this team [26]. Additionally, we identified and validated antimicrobial-resistant genes (AMR) using an amplicon sequencing approach in this study. We further evaluated the bacteriophage content of the human skin virome to identify factors that confer stability of the acquired gene content.

## Materials and methods

### Dataset acquisition and virome analysis for auxiliary metabolic genes

In addition to 42 previously sequenced subjects presented in Graham et al. [26] (SRA project PRJNA754140) (n = 642), skin viral metagenome samples from 18 study participants (n = 80) were collected and processed as described previously [26]. We sampled study participants at three different anatomical locations (right hand, left hand, and scalp) across five time points spanning a six-month period post the initial collection time point using a tandem wet-dry swab technique [26]. Viral particle enrichment was performed by filtration through a 0.22 µm filter to remove cellular and bacterial contaminants but no DNAse treatment was used due to low viral yield. The resulting viral particles were extracted using the QiAmp Ultra-Sensitive Virus Kit (Qiagen, Hilden, Germany) according to the manufacturer’s protocol. Viral DNA was amplified using the TruePrime whole genome amplification (WGA) Kit (Syngen Biotechnology, Inc, Taipei City, Taiwan). Library preparation for sequencing was performed using the NEBNext Ultra II Library preparation kit (New England Biolabs, Ipswich, MA, USA) according to the manufacturer's protocol. The resulting libraries were sequenced using the Illumina HiSeq platform using the 250 bp paired-end sequencing strategy.

All sequencing reads were quality filtered and processed to remove host contamination, as described in Graham et al. [26]. Bacterial contamination was assessed by mapping quality filtered reads to the Silva 16S ribosomal database v.138.1 using BBMap parameters described for high precision mapping of contamination as suggested in the BBMap Guide [27]. Ninety-six-point five percent (96.5%) of the samples contained less than 0.2, 16S rRNA reads per thousand reads per sample and were thus considered to be enriched for viral sequences with negligible bacterial contamination based on the criteria described by Roux et al. [28] (Additional file 1: Table S1).

Using MEGAHIT v.1.2.8 [29], a dual assembly approach was employed to assemble the quality filtered viral reads. Assembly datasets consisted of a master meta-assembly using all reads, and an assembly dataset of assemblies performed within each sample. The same bioinformatic assessment of samples was performed on the negative control samples, as described in Graham et al. [26]. A meta-assembly was constructed using all negative control reads and sample contigs from both assembly datasets were mapped to the negative control contigs greater than 1 kb using BWA-mem [30] to remove reads resulting from contaminants. Unmapped viral contigs greater than 1 kb were retained for further analysis.

### Bacteriophage annotation, diversity, and taxonomic classification

Contigs larger than 1 kb were retained and analyzed using the VIBRANT (Virus Identification By Iterative Annotation) package [31] (see Additional file 2: Table S2 for V-score information). VIBRANT phage outputs were used to identify auxiliary metabolic genes present within the skin virome and to identify persistent auxiliary metabolic genes across samples. The contigs identified through VIBRANT as having bacteriophage origin were taxonomically classified using Kaiju v.1.7 [32] and used for subsequent analyses.

To identify abundance of auxiliary metabolic genes in phages, quality filtered reads were mapped to VIBRANT identified bacteriophage contigs to determine abundance. Read mapping was performed using Bowtie 2 v.2.3.5 [33] and SAMtools v.1.9 [34] to identify the abundance of each viral contig within each sample. The resulting read counts were further analyzed using the Phyloseq package in R v.3.6.3 [35]. Diversity analysis of viruses and AMG containing bacteriophage populations was performed using the Phyloseq object table created using the read count abundance and metadata.

### AMR gene identification in bacteriophage contigs

Bacteriophage contigs containing all phage types generated via VIBRANT [31] were validated using the Blast-N algorithm [36] against a custom-built Blast database generated using the MEGARes v.2.0.0. AMR gene database [37] was used to identify AMR genes within the viral contigs identified. Filtration criteria of e-values < 1 × 10^–3^ with greater than 40% AMR gene coverage, > 80% nucleotide synteny, and bit scores greater than 70 were used to identify true AMR gene hits, as suggested by Enault et al. [38]. All contigs were mapped to AMR genes present in the MEGARes v.2.0.0. AMR gene database [37] using alignment program Bowtie 2 v.2.3.5 [33] and SAMtools v.1.9 [34] to obtain AMR gene abundance count data for each sample.

### Statistical analysis of bacteriophage diversity and stability

Alpha diversity of bacteriophage viral contig abundance identified by VIBRANT was assessed using the observed, Shannon, and inverted Simpsons alpha diversity metrics [39, 40]. A one-way repeated measures model with Shannon diversity metrics being used as the dependent variable was used to evaluate the overall stability of the bacteriophage population over time. To evaluate changes within-subject (intrasubject) variation over time in the phage community composition, the variable “subject” was used as a fixed effect with repeating measures of “time”. A one-way repeated measures ANOVA was performed to establish if the predicted phage diversity of a study participant significantly changes over time.

A Bray–Curtis dissimilarity matrix was generated and visualized using a principal coordinate analysis (PCoA) plot for Beta diversity analysis. Permutations of ANOVA (PERMANOVA) were performed using the Adonis test in the R package Vegan v.2.5–7 [41] to assess the effect of bacteriophage changes in beta diversity across the study participants. This was done to determine if the skin bacteriophage community of a study participant was significantly different from another subject’s bacteriophage community (i.e., intersubject variation). Alpha and Beta diversity was assessed across the bacteriophage count data.

The total abundance for each AMR gene containing contigs was evaluated by summing across time points for each anatomical location within a subject. Additionally, contigs containing specific AMR genes were statistically analyzed for temporal changes over time within-subject (intrasubject) and between-subjects (intersubject) using Freidman’s test utilizing an unweighted diversity matrix using a repeated measures generalized linear model. This test was a generalized linear mixed model with subject as a fixed effect to emulate repeated measures and a zero-inflated negative binomial distribution (using the glmmADMB package) for AMR genes with inflated zero counts [42].

### Amplicon sequencing of the erm(C) gene diversity

Before whole genome amplification from all 60 individuals, viral DNA samples were subjected to amplicon-based sequencing of the *erm(C)* gene (n = 704). Using primers specific to *erm(C)* (Forward 5′ AATCGTCAATTCCTGCATGT; Reverse 5′ TAATCGTGGAATACGGGTTTG) [43], a 299 bp region of the *erm(C)* gene was amplified and sequenced using the sequencing strategy described by Koztch et al. [44]. PCR reactions contained 1X Terra™ PCR Direct Polymerase Mix, 0.5U of Terra Taq polymerase (Takara Bio, Kusatsu, Shiga, Japan), and 1–2 ng of DNA. The PCR cycle included: initial denaturation of 98 °C for three minutes, followed by 30 cycles of 98 °C for 30 s, 55 °C for 30 s, and 68 °C for 45 s. Following the 30 cycles, a final extension of 68 °C for 4 min was performed. The resulting *erm(C)* amplicon products were visualized using 1.5% agarose gel electrophoresis to ensure amplification of the correct product size. Only samples that contained the correct fragment size were used for subsequent processing and sequencing. Samples were normalized using Norgen’s (Norgen Biotek Corporation, Canada) NGS Normalization 96-well Kit, following the manufacturer’s procedure recommendations using 10 µl of PCR product per sample and eluted in 20 µl. After normalization, 10 µl of each sample was pooled and concentrated using the NucleoSpin Gel and PCR clean-up kit (Macherey–Nagel, Düren, Germany) and associated PCR clean-up protocols. The concentrated libraries were further purified using a 2% agarose gel, and bands consistent with the correct amplicon size were cut from the gel to reduce primer-dimer contamination in the library samples before sequencing. Library DNA was purified using the NucleoSpin Gel and PCR clean-up kit (Macherey–Nagel, Düren, Germany) using described protocols for agarose gel PCR clean-up. Each sequencing library's nucleotide length (bp) distribution was assessed using an Agilent 2100 Bioanalyzer (Agilent Technologies, Inc, Santa Clara, CA, USA) with high sensitivity DNA chips to identify sample base pair distribution and sample concentration. Additionally, libraries were quantified using the DeNovix dsDNA High Sensitivity Kit (DeNovix, Inc, Wilmington, DE, USA). The libraries were then sequenced using the 250 bp paired-end sequencing strategy on the Illumina Miseq platform (Illumina, Inc, San Diego, CA, USA). Three blank negative controls containing PCR-grade water were assessed using the same protocol for every plate amplified and sequenced. Additionally, negative control samples from sample collection and extraction procedures were also assessed. All negative and blank controls did not produce any amplicons during the post-amplification and gel electrophoresis steps, nor did they produce any *erm(C)* positive sequencing reads.

### Erm(C) sequence variation and abundance

The quality filtered reads (quality threshold of Q30 or greater) were analyzed using the DADA2 pipeline [45]. To identify *erm(C)* reads, a custom Blast database was produced using all *erm(C)* gene sequences provided in the MEGARes v.2.0.0. database [37]. All sequence variants identified using DADA2 were Blasted to the custom *erm(C)* gene MEGARes database. Sequences that did not have a positive Blast hit with > 70% nucleotide sequence identity to an *erm(C)* gene were removed from the dataset, thus retaining only positive *erm(C)* sequence variants.

The quality filtered Amplicon Sequence Variants (ASVs) were aligned using MUSCLE v.3.8.1551 [46] to obtain a multiple sequence alignment to compare nucleotide differences between ASVs. Alignments were trimmed to retain similar sequence lengths, and a phylogenetic tree of the variants was generated using IQ-Tree v.1.6.12 [47] under the best fit HKY + F + G4 substitution model as determined by IQ-Tree. The resulting phylogenetic tree was visualized with iTOL v.6.5.2 [48].

Conformational and tertiary protein mutagenesis due to nucleotide polymorphisms were identified using the protein variant structural effect prediction tools PROVEAN (Protein Affect Variation Analyzer) [49], SIFT (Sorting Intolerant from Tolerant) [50], and Missense-3D [51]. Heatmaps associated with functional viability of the *erm(C)* gene as determined using the protein variant effect tools were produced using iTOL [48]. 3D visualization of polymorphic regions on the *erm(C)* monomer were generated by mapping variants to pre-established 3D models of the *erm(C)* gene using the SWISS-MODEL Workspace [52] and associated *erm(C)* reference models present in the SWISS-MODEL Repository [53].

## Results

### Human skin virome bacteriophage diversity and temporal stability

**Fig. 1:**
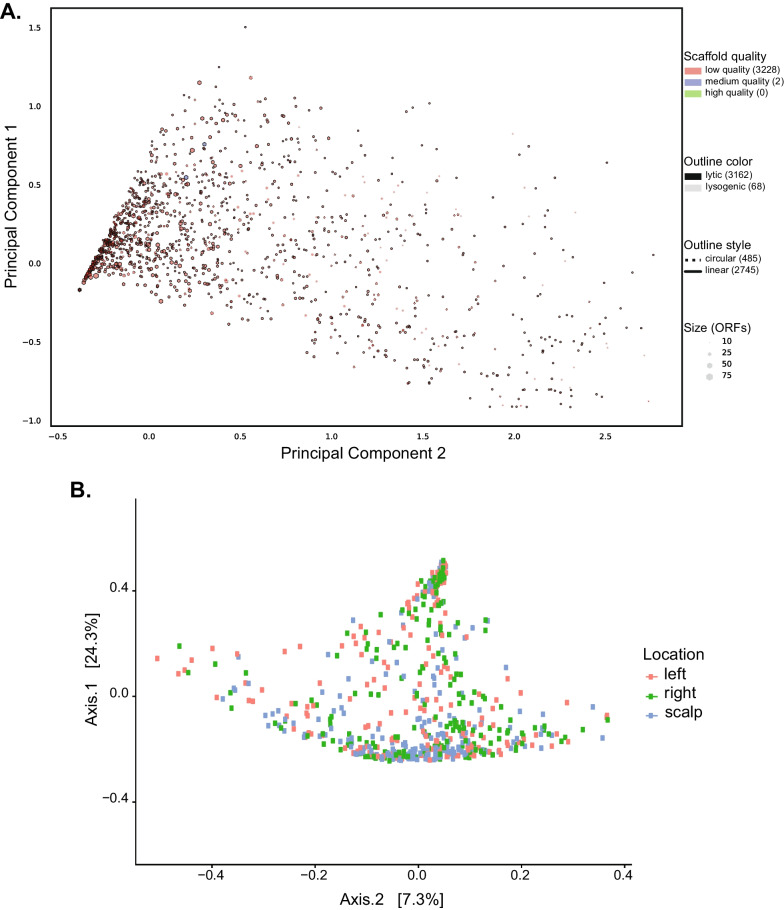
The human skin bacteriophage virome is composed of viral communities with diverse phenotypic and ecological characteristics. Bacteriophage contigs were identified from a metagenomic meta-assembly of human skin virome samples collected from subjects. **A** Principal component analysis shows annotated bacteriophage genome variation of the 3230 contigs of 1 kb length and longer that were identified as bacteriophage, with 3162 contigs being lytic phage and 68 being lysogenic phage. Of the identified phage, 485 were identified as having a circular conformational genome, and the remaining 2745 phage assemblies had a linear conformation genome. **B** Bacteriophage contig beta diversity differences are driven by anatomical skin location sampling site and subject differentiation. PCoA plot of Bray Curtis dissimilarity distances of virome samples by location to assess VIBRANT identified bacteriophage beta diversity across sampling site locations. β-diversity clustering was assessed using a PERMANOVA on reported Bray Curtis dissimilarity distances, and it was found that clustering based on both variables subject (*P* = 0.001, R2 = 0.15102) and location (*P* = 0.001, R2 = 0.13965) were significant

Data from our previous study (n = 624) [26] was combined with 80 additional samples from 18 new study participants to evaluate bacteriophage diversity and abundance in the human skin virome. The resulting contigs greater than 1 kb were analyzed to identify contigs that may have originated from bacteriophage. We identified 3230 contigs, > 1 kb in length, to be of bacteriophage origin using VIBRANT. This included 3162 contigs from lytic phage and 68 contigs from lysogenic phage. Of the identified phages, 485 had a circular conformational genome, and the remaining 2745 phage contigs were presumed to have a linear, conformational genome (Fig. 1A). When the raw sample reads were mapped back to the assembled contigs, we determined that the phages identified using VIBRANT from each individual represented between 5 and 57% of the total skin virome reads per sample. This lower annotation of viral reads combined with very low contamination of bacterial sequences in our meta viromes and bioinformatic filtering of human genome contaminants suggests that current databases poorly represent viral phage diversity, and the human skin virome carries a more extensive diversity of viruses than previously described [17–19].

Initial diversity assessments were performed in a reference-independent manner treating each contig as its own viral classification to reduce reference bias. Alpha diversity of the assembled contigs represented by the Shannon diversity index displayed gender and anatomical location within-subject (intrasubject) to be not-significantly different (*P* = 0.6481 and *P* = 0.0923, respectively). However, alpha diversity across subjects (intersubject) was highly significant (*P* = 1.68 × 10^–8^). Additionally, a significant difference in alpha diversity within-subject over time was observed (*P* = 0.0048).

Beta diversity was evaluated using a Bray–Curtis dissimilarity matrix and visualized using a PCoA plot (Fig. 1B). Clustering by sample location (i.e., scalp, right hand, left hand) was evident in the plot. Using the distances generated in the Bray–Curtis dissimilarity matrix as the dependent variable, a PERMANOVA was performed. All variables tested had a significant impact on viral diversity changes with 15% (R^2^ = 0.15102) of the variation explained by subject-to-subject variation (*P* = 0.001) and 14% (R^2^ = 0.13965) explained by location within a subject (*P* = 0.001). Beta diversity was evaluated using Jaccard dissimilarity distances for binary presence and absence of each phage contig within the study population. Similar results were observed with Jaccard dissimilarity distances where 10% (R^2^ = 0.10668) of the variation in phage diversity was explained by subject-to-subject variation (*P* = 0.0001) and 13% (R^2^ = 0.13975) was explained by anatomical location within-subject (*P* = 0.016).

**Fig. 2:**
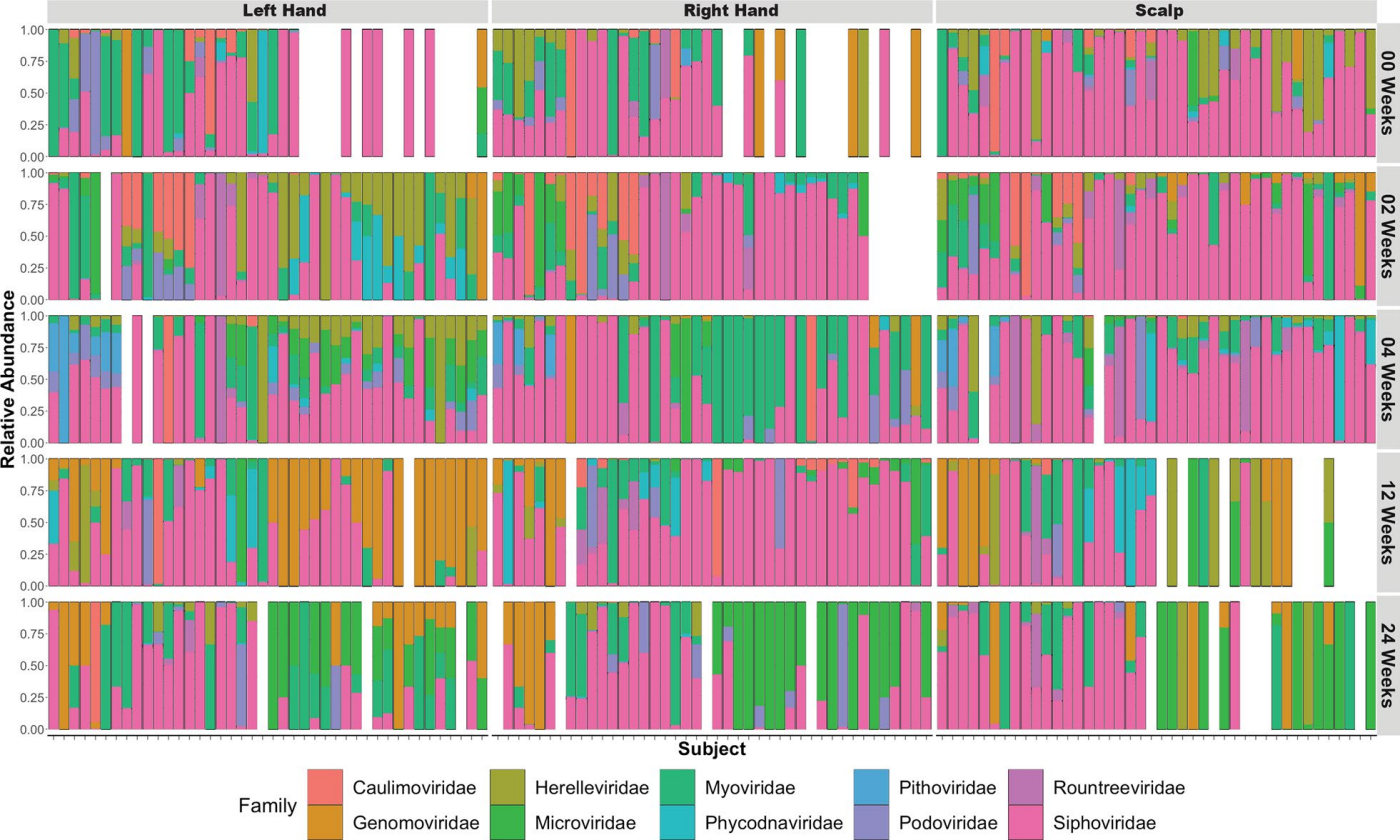
Taxonomic diversity and relative abundance of annotated human skin associated viral communities across subjects by anatomical location over time. Taxonomic designations are representative of viral family classifications of annotated contigs. Contigs that could not be classified at a family level are not shown. A table with proportion of unclassified viruses is shown in Additional file 3: Table S3. Each bar represents a single sample from a subject. Bars are separated by sampling anatomical skin site as noted on top of the figure and time post initial (00 Weeks) sampling collection timepoint. Gaps in figure columns are attributed to missing data from subjects who did not complete sampling

The largest proportion of the phages identified that could be taxonomically classified were classified into the Class *Caudoviricetes*. Of those that could be classified, viruses with morphological features of *Myoviridae* or *Siphoviridae* were predominant (Fig. 2). Consistent with common commensal human skin bacteria, most of the phages classified were phages known to infect *Staphylococcus* species. However, only approximately 16% of the total phage contigs were able to be taxonomically classified using current viral reference databases. The remaining unclassified contigs consisted of sequences representing putatively novel phages. Because a high percentage of phages were unclassified, this justified utilizing database-independent approaches to evaluate the human skin phage diversity in subsequent ecological and diversity analysis.

### Auxiliary metabolic genes found in the human skin virome

**Fig. 3:**
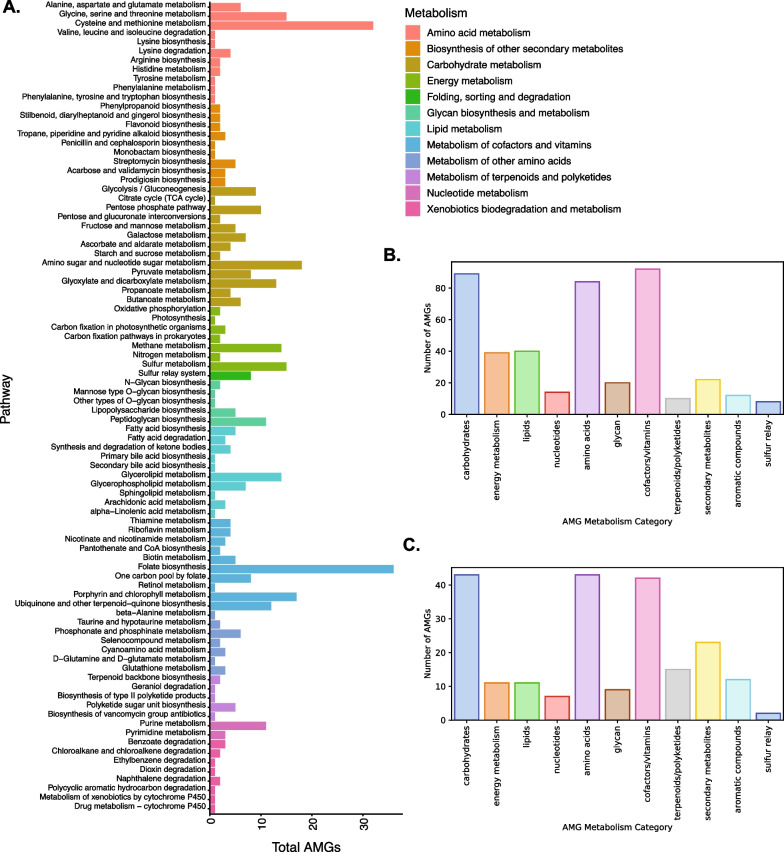
Nonredundant bacterial auxiliary metabolic categories identified in human skin virome bacteriophage contigs. Overall metabolic categories showed similar count ratios in both the within-sample assembled bacteriophage dataset (**B**) and the meta-assembled bacteriophage dataset (**C**). Each metabolic category was further broken down by AMG-associated KEGG pathways for the meta-assembled dataset (**A**). Counts are representative of the total number of nonredundant AMGs present within each category identified within the generated bacteriophage assembly databases and are not a measure of abundance within the study population. The persistence and abundance of each metabolic category and metabolic pathway are described in Additional file 4: Figs. S1 and S2

Identified bacteriophage contigs were assessed for the presence of bacterial host-specific auxiliary metabolic genes (AMGs). A total of 648 AMG genes were identified using VIBRANT within contigs identified as bacteriophages. The annotation and distribution of AMGs, as defined by their KEGG [54] annotation, are described in Fig. 3B–C. The greatest diversity in the overall categories of AMGs was observed in genes involved in carbohydrate metabolism, amino acid synthesis, and cofactors and vitamins. Each metabolism category was broken down further by the metabolism pathway and is shown in Fig. 3A. The pathways most associated with AMGs included cysteine and methionine metabolism and folate biosynthesis. The overall abundance and persistence of each metabolism category across all five time points by subject and location are shown in Additional file 4: Figs. S1 and S2. Abundance and persistence were only assessed for the meta-assembly dataset containing only subjects with complete collection sets (i.e., for 42 out of the 60 subjects) due to the possibility of missing time points affecting abundance. As shown in Additional file 4: Figs. S1 and S2, the abundance of each metabolic pathway varied by subject and location within-subject. AMGs involved in lipid metabolism, protein folding and sorting, amino acid metabolism, and metabolism of cofactors and vitamins were the most abundant within the population for certain subjects (Additional file 4: Fig. S1).

Upon further inspection of the AMGs involved in the biosynthesis of secondary metabolites and biosynthesis of terpenoids and polyketides, it was noted that certain identified AMGs were involved in biosynthesis pathways of production of antibiotics such as penicillin, cephalosporin, vancomycin, and streptomycin (Fig. 3A). However, these AMGs were not persistent over time in the sampled population (Additional file 4: Fig. S2). The presence of AMGs within the skin bacteriophage population is notable as the production of these antibiotics could potentially affect bacterial community structure and function, leading to increased fitness of the bacterial host.

### Antimicrobial resistant genes in the human skin bacteriophage population

To identify AMR gene presence and abundance in the bacteriophage contigs, all viral contigs were compared using Blast-N to a custom database generated using the MEGARes v.2.0.0. AMR gene database [37]. Blast results were filtered to only contain hits with recommended cutoffs for accurate AMR gene identification [38]. Sixteen contigs were identified that contained at least one AMR gene that passed the cutoff recommendations (Table 1). These included AMR genes, *rlm(H)*, *par(E)*, *par(EF)*, *par(C)*, *msr(D)*, *msr(E)*, *mef(A)*, *fus(B)*, *mph(E)*, and *erm(C)*, that encode antibiotic drug resistance; *mer(B)* and *mer(G)* which encodes mercury resistance; and multi-compound drug and biocide resistance gene *qac(F)*. Alignment information, e-value, coverage, and percent identity information for all AMR genes identified are reported in Table 1. It was noted that contigs contained more than one AMR gene, such as those containing both *mef(A)* and *msr(D)*. In addition to contigs containing dual *mef(A)* and *msr(D)* genes, one additional phage contig contained an *msr*-related gene of *msr(E)* and two copies of the phosphotransferase *mph(E)* gene.

**Table 1:**
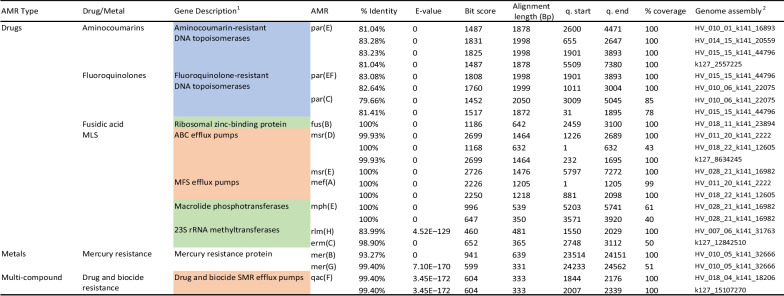
Antimicrobial-resistant gene presence in bacteriophage metagenome assemblies

^1^Gene description color is associated with AMR mechanism or resistance type

^2^Genome assembly nomenclature is consistent with given contig names in assembly fasta files. Names beginning with HV are from within sample assemblies, and those beginning with k127 are from the overall meta-assembly dataset

### Temporal stability of AMR erm(C) gene in the human skin virome

The erythromycin resistance gene *erm(C)* was the most abundant AMR gene and was highly abundant in certain subjects across multiple anatomical locations and time points. To assess *erm(C)* gene temporal stability within the population, a repeated measures generalized linear mixed model was used over time across subjects (Additional file 4: Fig. S3) to evaluate the abundance and persistence of *erm(C)* genes. This analysis identified *erm(C)* abundance to be stable over time as suggested by there being no significant change in abundance of *erm(C)* over time (*P* < 0.05 = 0.734). Using the Friedman test, it was determined that there was no significant difference in abundance over time within-subject (intrasubject) (*P* < 0.05 = 0.216808).

**Fig. 4:**
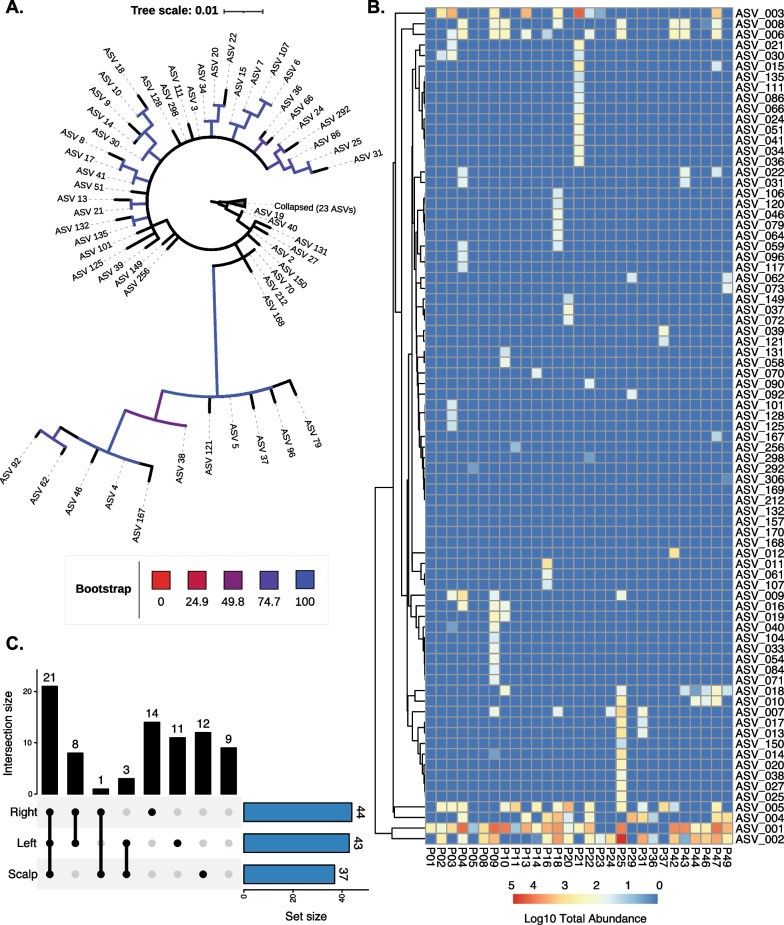
*Erm(C)* variant phylogeny and diversity across three anatomical locations 60 individuals over 5 time point collections spanning 6-months. **A** Maximum likelihood phylogenetic analysis of *erm(C)* nucleic acid sequence variants. ASVs were aligned using MUSCLE v.3.8.1551 [46], inferred using the best-fit substitution model of HKY + F + G4 as determined by IQ-Tree [47], and visualized using iTOL [48]. Branch bootstrap support is gradient colored, with red representing 0% support and blue representing 100% support. Tree was rooted using ASV_1 which has 100% sequence similarity to reference *erm(C)* sequence [WP_001003263.1]. Phylogeny of variants shows distinct evolutionary subpopulations. Many of the variants shared high sequence similarity and thus were clustered together. Full tree without clustering is available in the (Additional file 4: Figs. S1 and S2). **B** Log10 total abundance of *erm(C)* ASVs summed across all time points and anatomical locations for a subject. Subject notation is indicated at the bottom. **C** Distribution of *erm(C)* variants across the three anatomical skin sites. Intersection size represents the number of ASVs that were present at the combination of locations, as noted by the scatter plot below the bar. Bars shown in blue (Set size) are the overall number of ASVs for each location

Though the presence of *erm(C)* in phage populations is stable, the abundance and the variation of the *erm(C)* within populations are poorly understood. Additionally, current studies have failed to identify (1) if variations in the gene exist, (2) if dominant variations are present within the microbiome, and (3) if different variations of the gene are present among different viral strains. To assess *erm(C)* functionality and genetic variation within the skin virome, we performed targeted paired-end amplicon-based sequencing of a monomeric region of the *erm(C)* gene in all 60 subjects (n = 894). The resulting reads were binned to identify amplicon sequence variants (ASVs) for the *erm(C)* gene. All sequence variants were then compared against the MEAGARes database [37]. Sequence variants that did not have high similarity to the *erm(C)* gene were removed, which resulted in 79 variants with ASV_1 having 100% nucleotide and amino acid sequence similarity to the reference *erm(C)* protein (WP_001003263.1). All *erm(C)* ASVs identified were aligned and phylogenetically compared with trees representing their phylogeny in Fig. 4A and Additional file 4: Fig. S4. Some *erm(C)* variants were present across all three skin anatomical locations and at all time points. In contrast, others were only present in certain locations, as shown in Fig. 4B–C.

**Fig. 5:**
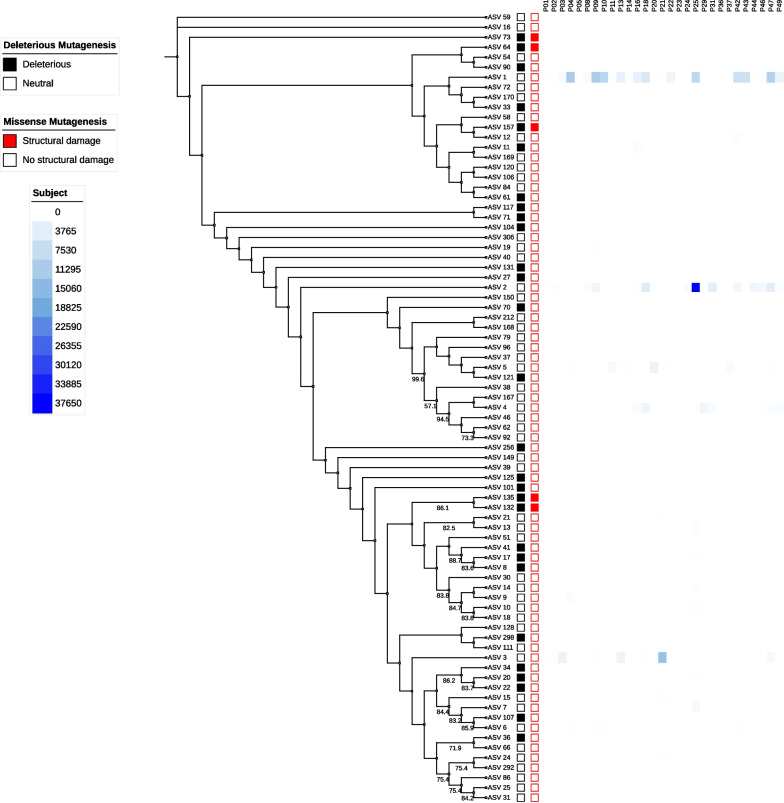
*Erm(C)* variant phylogeny (unscaled) shows a high abundance of only functional versions of *erm(C)* across subjects. Protein folding structural damage and deleterious effects from mutagenesis within the sampling population were predicted using the protein variant structural effect prediction tools PROVEAN, SIFT, and Missense-3D [49–51]. Squares to the right of the variant id (i.e., ASV ##) indicate the presence of a negative impact on protein functionality via either deleterious mutagenesis (black square) or structural damage (red square). A heatmap representing total abundance for each *erm(C)* sequence variant by subject was also provided. Only variants that did not exhibit deleterious or structural damage due to mutagenesis displayed high abundance across subjects

**Fig. 6:**
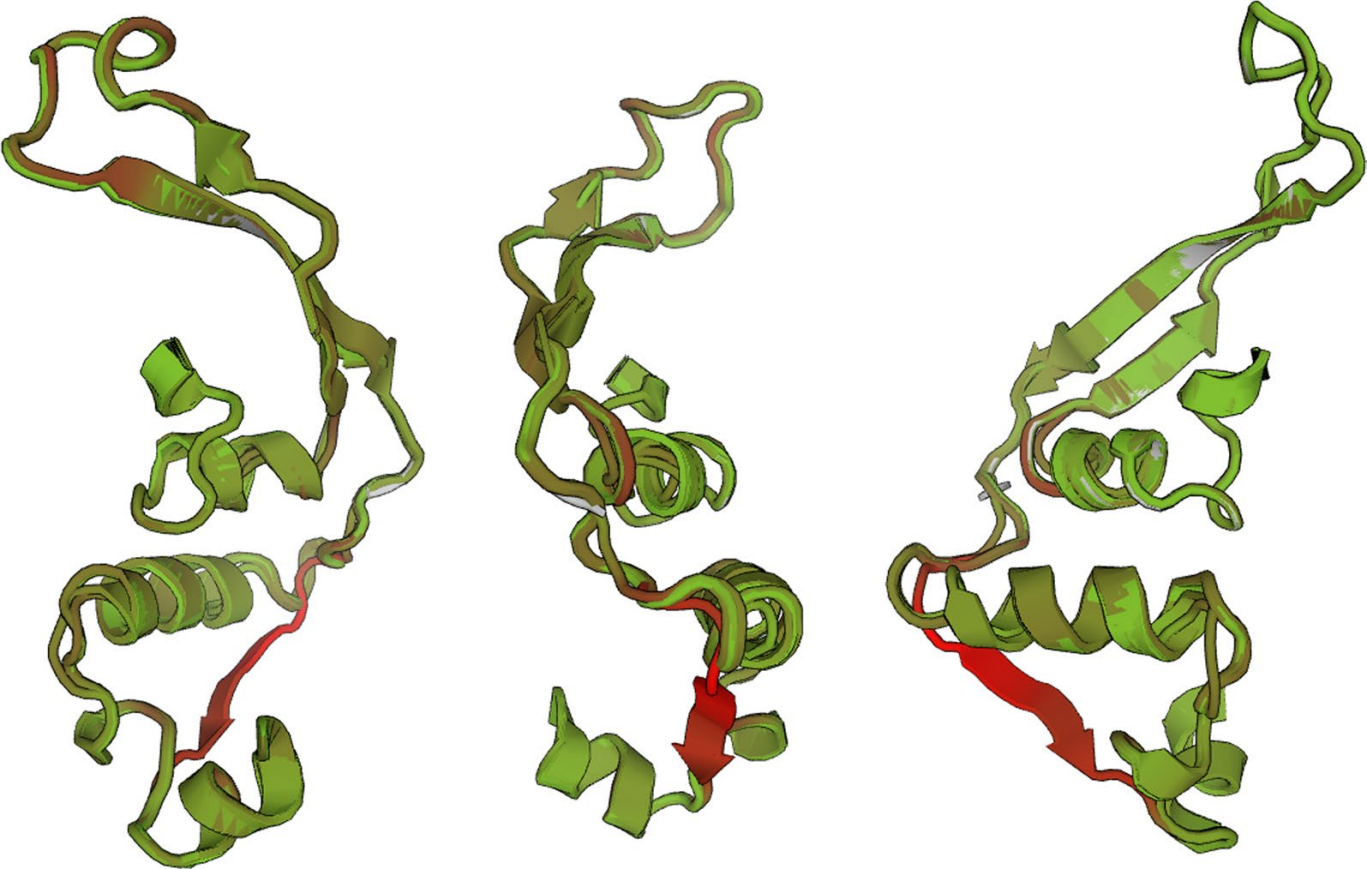
3D model of sequenced *erm(C)* monomeric region variant tertiary protein folding mutagenesis. Models for each of the 79 identified *erm(C)* sequence variants were produced in the SWISS-MODEL Workspace [52] by mapping variant amino acid sequences to pre-established 3D models of *erm(C)* in the SWISS-MODEL repository [53]. Regions of confirmational variation are indicated in red, and regions of structural conservation across the sequenced monomer are indicated in green

To investigate if functionality is associated with the abundance and persistence of *erm(C)* sequence variants, all variants were translated, and their amino acid sequences were assessed using protein mutagenesis prediction tools [49–51] to establish potential point mutations that putatively affect the function of the *erm(C)* protein. Predicted deleterious mutations and missense mutations that putatively result in structural damage to the protein were identified in silico. Structural damages identified mainly consisted of substitutions resulting in the expansion of the protein cavity (Additional file 4: Figs. S5 and S6). An additional substitution at the W19R region of the protein monomer resulted in a replacement of a buried hydrophilic charged residue with a hydrophilic uncharged residue in one ASV (Additional file 4: Fig. S7). Though not necessarily functionally detrimental, as determined using in silico predictions, missense mutations and deletions resulted in structural variants of the *erm(C)* that presented differing protein folding conformations, such as the conversion from alpha-helices to beta-sheets (Fig. 5). All highly abundant sequence variants displayed the ability to synthesize a functional protein, whereas the low abundance ASVs contained mutations that resulted in putatively non-functional proteins (Fig. 6).

## Discussion

Viruses play vital roles in shaping microbial communities by improving host fitness, controlling bacterial populations through predation, and helping increase metabolism by overcoming metabolic bottlenecks [4, 6, 17, 55, 56]. For instance, over time, filamentous bacteriophages have acquired numerous host-specific genes that have allowed them to have symbiotic relationships with their bacterial hosts, thus promoting host survival and the production and spread of phage progeny [6]. The retention of host genes and the metabolomic influence of phages on bacterial hosts have been described in aquatic communities [13, 15], and human and animal gut microbiomes [25, 31, 57].

Studies are limited that describe phage diversity and ecology in humans other than the gut [17–19, 26, 58–66]. To date, only a few studies have addressed the human skin virome and its taxonomic composition [17–19, 26, 59, 62]. Additionally, studies describing AMR genes in the skin virome and other auxiliary genes are limited [17]. Here we describe bacteriophage diversity, AMGs, and AMR genes present in the human skin virome and possible roles of the human skin virome using temporal information from five time points spanning six-months across three anatomical skin locations (left hand, right hand, and scalp) from 60 human participants.

We identified 3230 bacteriophage contigs from human skin viral metagenomes. Bacteriophage contigs identified in this study predominantly originated from the viral Families *Myoviridae* and *Siphoviridae*, which both belong to Order *Caudovirales*. Previous studies have reported that the skin virome consists primarily of *Caudovirales* bacteriophages [17, 18, 26, 62]. While our study is consistent with previous reports, current viral reference databases consist of a disproportionate amount of *Caudovirales* viral genomes, and this fact could contribute to an annotation bias in virome studies, ours included. This is further reflected by the fact that only 16% of the putative phage contigs we assembled could be taxonomically classified using current reference databases. This fact underscores the lack of knowledge regarding phage diversity in the human skin virome. Therefore, we speculate that most of the phages that we have identified in this study are novel phages. Additional work assessing bacteriophage identification, isolation, annotation, culture, and comparative genomics is needed to fully understand bacteriophage taxonomic composition and diversity in the human skin virome. However, our filtration using 0.22 μm filters may have resulted in removal of large viruses from the skin virome and may be lacking large viral particles. Additionally, the whole genome amplification used in this study may have led to amplification bias.

Though many studies have shown the presence of host-specific genes such as AMR genes in *S. aureus* and that transduction via *S. aureus* phage is a common form of horizontal gene transfer of these AMR genes, very few culture-independent studies have been done that look at the human skin virome and its composition and abundance of host-related genes in phages [17]. Studies have investigated the skin virome composition and the temporal stability and diversity of the virome; however, many of these works did not address the presence of AMG or AMR genes or were smaller-scale studies with lower sample numbers with short sampling periods of only a couple of weeks without repeated sampling periods [17–19, 26, 62].

The acquisition of bacteria-specific genes, especially genes associated with host immune defense evasion and host genes associated with increased replication or cell proliferation to aid in viral replication and spread, is not a new concept in virology [4, 17, 31, 67, 68]. However, it is crucial to understand what genes are enriched in viromes and how expression of such genes could affect host function and persistence. To this end, our phage contigs contained 648 AMGs within the human skin virome. This study identified the human skin virome to carry genes associated with carbohydrate metabolism and amino acid synthesis. These genes are critical when the virus overtakes the cell for replication as carbohydrate metabolism could lead to increased energy for replication, and amino acid synthesis genes could help synthesize viral capsid during replication. Recently, it was shown that Severe Acute Respiratory Syndrome Coronavirus 2 (SARS-CoV-2) could use host folate and one-carbon metabolism to bolster replication [69]. Similarly, it may be possible that bacteriophages carry folate metabolism genes to increase de novo purine synthesis to increase viral replication. The presence of auxiliary metabolic genes associated with folate biosynthesis has been reported in rumen viruses and has been suggested in one-carbon metabolism [57]. As such, the increased folate metabolism genes may help utilize one-carbon substrates to provide energy during replication.

We also identified AMR genes associated with three main antimicrobial drug resistance mechanisms among the bacteriophages identified. This included efflux transport pumps found in gram-positive and gram-negative bacteria that help transport toxic compounds and antibiotic drugs out of the cell [70]. Of the five main families of efflux pumps associated with antibiotic resistance, genes encoding efflux pumps belonging to ATP-binding cassette (ABC), the major facilitator superfamily (MFS), and the small multidrug resistance family (SMR) were all identified within the phage population. We identified the MFS efflux pump protein *mef(A)* and the ABC efflux pump protein *msr(D)*, present on the same contigs. In *Streptococcus pneumoniae* and *Staphylococcus epidermidis*, when dually expressed, these two proteins act as a dual efflux pump that gives moderate resistance against 14- and 15- membered macrolides such as erythromycin, clarithromycin, and azithromycin [71]. Having the *mef(A)* gene alone results in high resistance levels to varying macrolide drugs and is considered a common gene associated with multidrug-resistant pathogens such as MRSA. The presence of the dual efflux pump of *mef(A)*-*msr(D)* (also commonly referred to as *mef(A)*-*mel*) confers a higher level of resistance to macrolides than that of just *mef(A)* alone [72]. Both *mef(A)* alone and dual *mef(A)-msr(D)* genes have been identified in prophage regions of bacteria and in phage genomes such as that of the *Streptococcus* infecting phages Tn1207.3 and Tn1207.1 [73–75]. The detection of dual efflux pumps within the phage population suggests that AMR genes can be moved across bacteria via phage-mediated transfection.

The efflux pump transcribing gene, *qacF*, was also observed in multiple viral contigs. These efflux pumps confer quaternary ammonium compound resistance and have been hypothesized to increase bacterial tolerance to antibiotics, especially antibiotics that inhibit cell wall synthesis [76, 77]. Bacteria containing *qacF* and *qac-*related genes have been reported in soil and agriculture related environments, including livestock related industries. The presence of this AMR gene in bacteriophages is not surprising since multiple study participants reported being in recent contact and working with livestock. This demonstrates that the phage diversity in the skin virome constantly changes and is, at least in part, acquired from the environment. Additionally, this suggests that mobile genetic elements may be a route in which AMR genes are horizontally transferred.

One of the most common AMR genes associated with antibiotic resistance is genes encoding proteins that directly interact with or modify bacterial ribosomes. This mode of action confers resistance to antibiotic agents that target transcription and translation. The 23S rRNA interacting methyltransferases *RlmH*, *erm(C)*, and phosphotransferase *MphE* were all identified in the phage population. These genes confer strong antimicrobial resistance to many drugs, such as aminoglycosides, macrolides, oxazolidinones, and streptogramins [70]. The presence of *erm(C)* in multiple contigs is of clinical importance since *erm(C)* is the best-studied resistance mechanism to MLSB (macrolide, lincosamide, and streptogramin B) in bacteria and is one of the leading AMRs associated with MRSA [71, 78]. Due to its high abundance within the population and its clinical relevance to human health, we investigated the abundance and distribution of the *erm(C)* gene within the study population using targeted amplicon-based sequencing to identify the SNP variation within the *erm(C)* gene to evaluate functional capabilities and phylogeny of this gene within our study population.

The *erm(C)* gene was highly abundant and temporally stable in some individuals. The stability of the *erm(C)* gene across the skin virome of study participants over a six-month period suggests that the gene plays an important role in phage stability and, in turn, bacterial host fitness. One can hypothesize that temporal stability and evolutionary retention of functional versions of AMR genes give associated phages an evolutionary advantage over viral strains that do not carry AMR genes or do not have functional versions of AMR genes. Thus, to establish the importance of AMR genes for persistence and to identify the evolutionary advantage of phages having AMR genes, we investigated the abundance and the presence of functional sequence variants of the *erm(C)* gene using the amplicon variants obtained through targeted sequencing. This analysis revealed that only functional variants of the *erm(C)* gene persist within the viral population at high abundance (Fig. 5). This suggests that *erm(C)* provides an advantage for the phages to persist in the human virome and demonstrates that phages can acquire multiple host genes that can impact microbiome community diversity and evolutionary selection, including genes that transcribe antimicrobial activity resistance.

## Conclusions

Viruses play an important role in modulating bacterial population and diversity. Here we investigated the human skin virome and skin associated bacteriophage population diversity, dynamics, and the auxiliary metabolic genes associated with these phages. Human skin viral metagenome samples revealed that the bacteriophage population on the skin is mainly composed of tailed bacteriophages in the viral order *Caudovirales*. Nevertheless, many phage contigs could not be classified due to the poor representation of human skin viruses in viral reference databases. We identified 648 different bacterial host-derived AMGs related to varying types of bacterial cell processes and functions. Additionally, we identified the antimicrobial-resistant genes *erm(C)*, *par(E)*, *par(EF)*, *par(C)*, *fus(B)*, *msr(D)*, *msr(E)*, *mef(A)*, *mph(E)*, and *rlm(H)*. These genes were, in some cases, subject-specific, whereas genes such as *erm(C)* were abundant across multiple individuals and were stable over time. This study demonstrates that the phages in the human skin virome carry auxiliary metabolic genes that increase host fitness and help with the persistence of the bacterial host and contribute greatly to bacterial-viral (phage) interactions. Findings from this study suggest that viral-host relationships are more complex than previously thought and highlight the importance of utilizing system-based approaches to study ecosystem interactions in order to fully understand microbiome diversity and function.

## Supplementary Information

**Additional file 1**. 16S reads per 1000 sequence reads in each sample.

**Additional file 2**. Total genes and v-score generated  by VIBRANT for KEGG, Pfam and VOG databases.

**Additional file 3**. Count data for viral abundance at family level.

**Additional file 4**. Supplementary Figures.

## Data Availability

Raw sequencing data is available through the NCBI Short Read Archive (SRA) under the project accession codes PRJNA754140 and PRJNA936861. Project metadata and bioinformatic pipeline scripts described in this manuscript are publicly available at: https://github.com/egrah3/Graham_2022_Virome_AMG_AMR.
