# Supplementary material for: The persistence and stabilization of auxiliary genes in the human skin virome
Source: Virol J. 2023 Mar 22;20:49. doi: 10.1186/s12985-023-02012-3 (PMC10031188; doi:10.1186/s12985-023-02012-3)
Supplement: Supplementary file 4 — Additional file 4. Supplementary Figures. [file 12985_2023_2012_MOESM4_ESM.pdf]

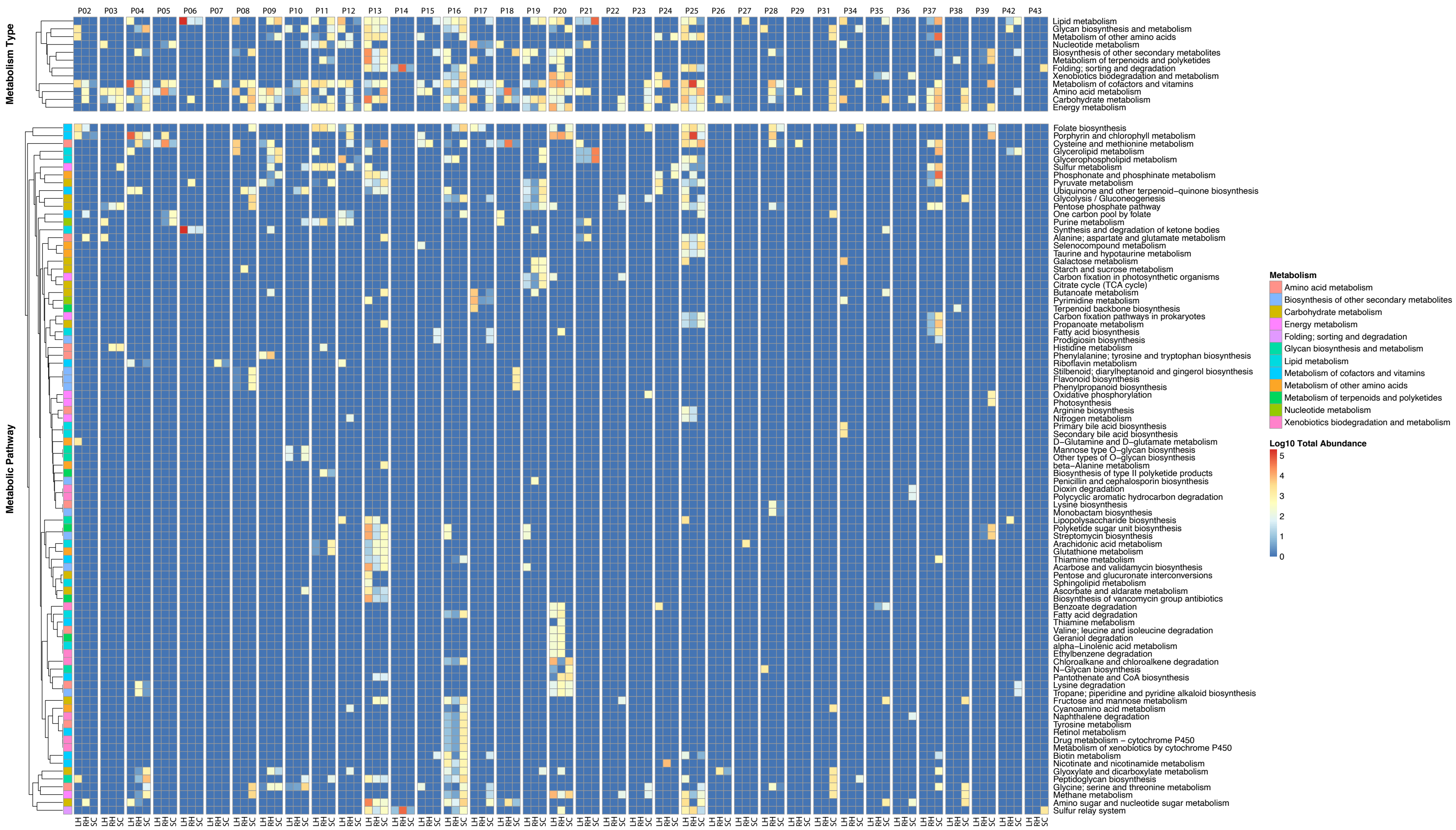

Supplementary Figure 1. Abundance (log10) of AMG metabolic categories and metabolic pathways by subject and location within-subject. Of the identified AMG bacteriophage, the abundance of each AMG grouping was summed across all time points by anatomical sampling location within a subject. A heatmap was then generated where each column is an anatomical location (LH = Left Hand; RH = Right Hand; SC = Scalp) which is separated by subject as denoted on top. Each row is associated with either a metabolic category (top heatmap) or a metabolic pathway within a category (bottom heatmap). For each heatmap, rows are clustered

based on AMG grouping log10 total abundance across the five time point collections similarity across the samples. The color-coordinated overall metabolism category is additionally noted in the leftmost column of the heatmap.

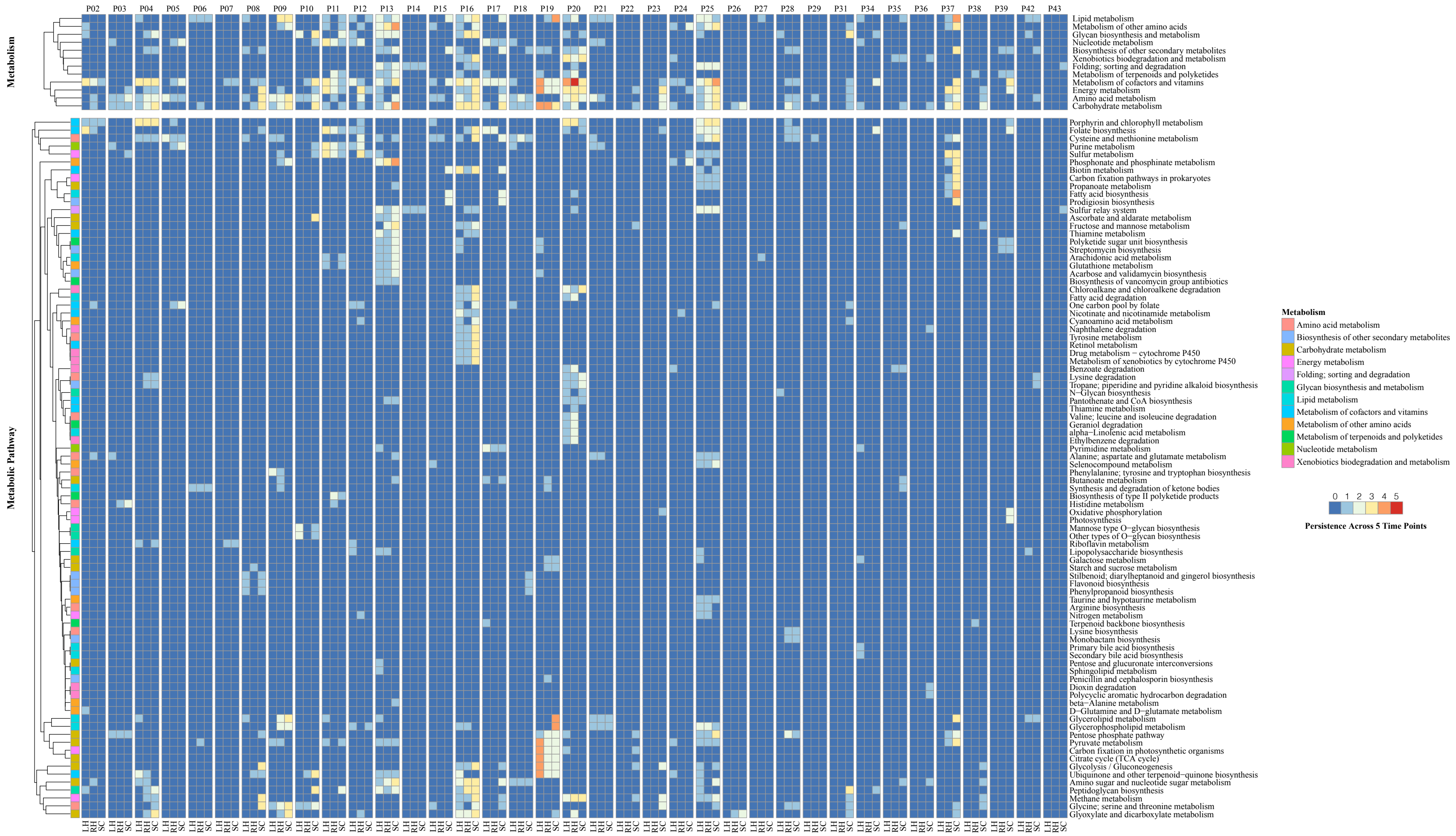

Supplementary Figure 2. Temporal persistence of AMG metabolic categories and metabolic pathways by subject and location within-subject. Each column is an anatomical location (LH = Left Hand; RH = Right Hand; SC = Scalp) which is separated by subject as denoted on top. Each row is associated with either a metabolic category (top heatmap) or a metabolic pathway within a category (bottom heatmap). Rows are clustered based on AMG grouping persistence across the five time point collections similarity across the samples. The color scale represents the persistence of the metabolic category or pathway in the five time points, with red

being present in five out of the five time points and blue being present in zero out of the five time points. The color-coordinated overall metabolism category is also noted in the heatmap's leftmost column.

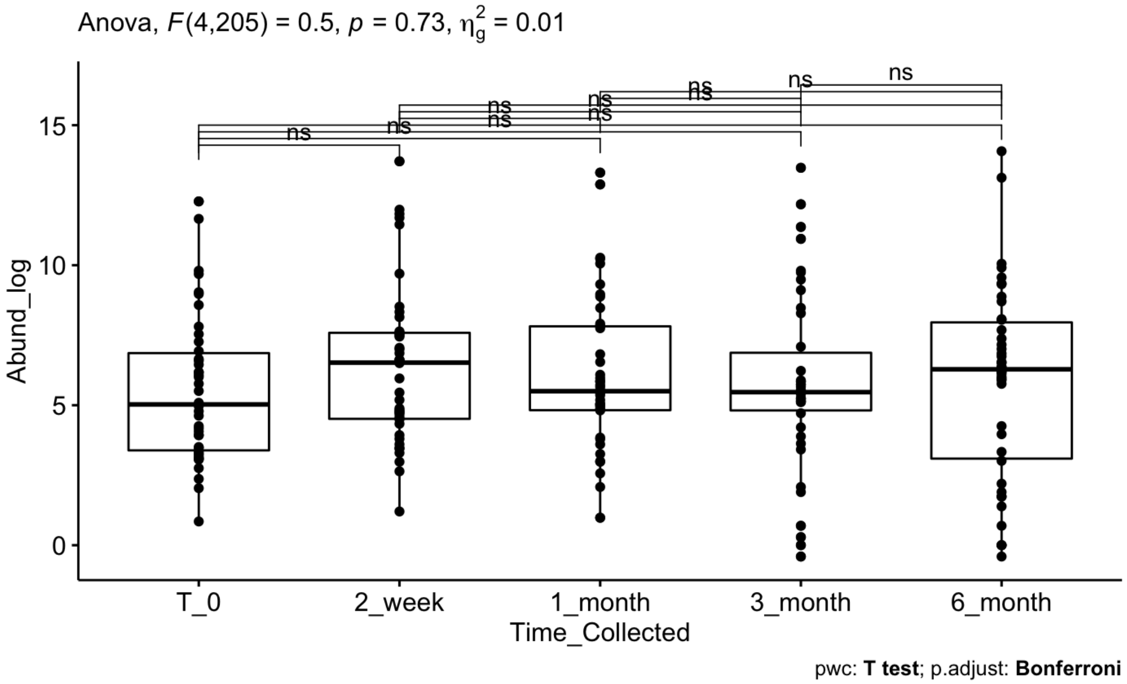

Supplementary Figure 3. Pairwise t-test of *erm(C)* abundance across subjects over time shows no significant difference over time. To test *erm(C)* stability over time within subjects, a pairwise t-test with a Bonferroni correction of the log abundance of *erm(C)* was performed in comparison to all time points within an individual. *Erm(C)* abundance within individuals was not significantly different over time (P=0.73).

ns nonsignificant at P>0.05; . significant at P¬=0.5; * significant at P<0.05; ** significant at P<0.01; *** significant at P<0.001; **** significant at P<0.0001

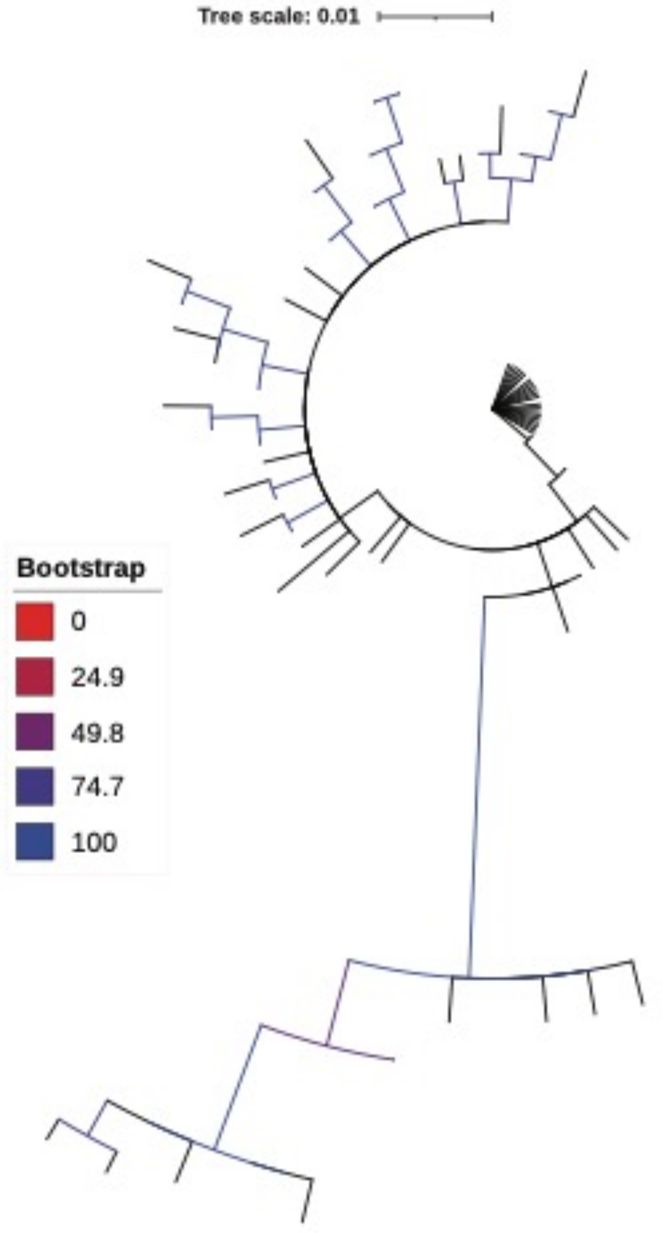

Supplementary Figure 4. Maximum likelihood phylogenetic analysis of *erm(C)* nucleic acid sequence variants. ASVs were aligned using MUSCLE v.3.8.1551 [46], inferred using the best-fit substitution model of HKY+F+G4 as determined by IQ-Tree [47], and visualized using iTOL [48]. Branch bootstrap support is gradient colored, with red representing 0% support and blue representing 100% support. Tree was rooted using ASV_1, which has 100% sequence similarity to the reference *erm(C)* sequence [WP_001003263.1].

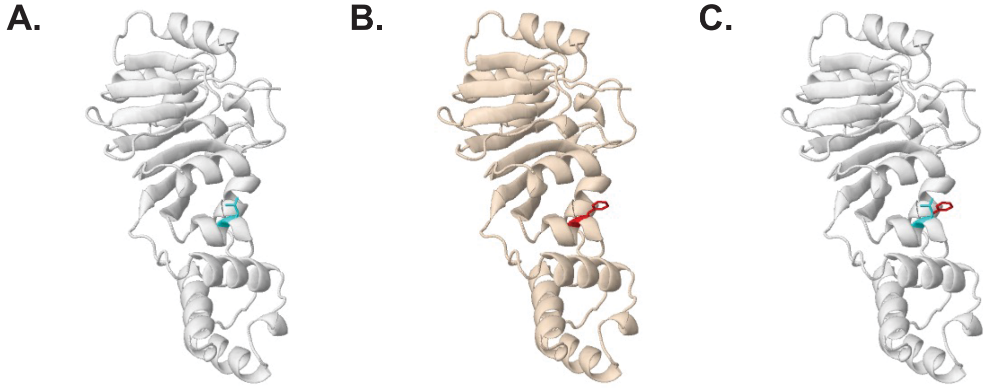

Supplementary Figure 5. Structural damage missense mutation identified in an ASV at the L142F region of the *erm(c)* gene as identified by Missense-3D [51]. The missense substitution detected in this ASV results in an amino acid change that leads to the expansion of the protein cavity volume by 105.624 $Å^3$. Location and 3D structure representation of the amino acid conformation is highlighted to show conformational changes in the protein structure of the *erm(C)* gene (A; highlighted in blue) due to a missense mutation (B; highlighted in red). An additional overlay of the original amino acid 3D structure and the altered amino acid structure is provided (C).

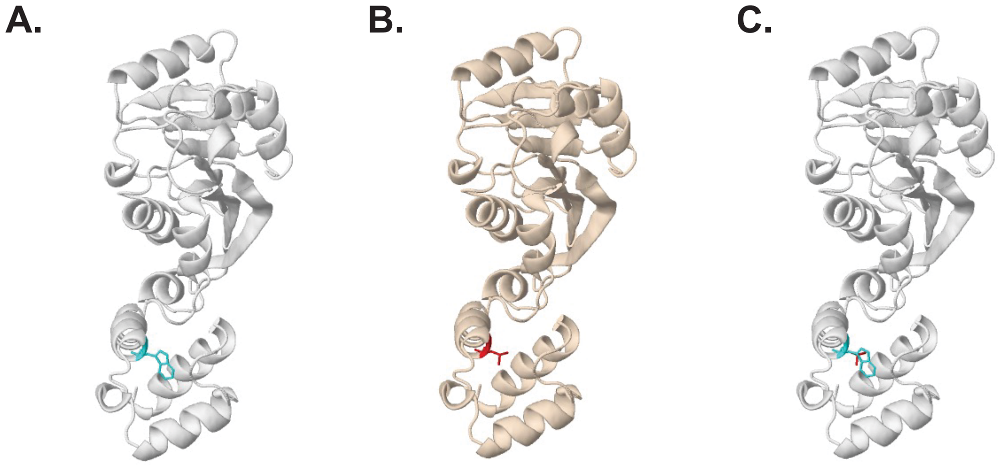

Supplementary Figure 6. Structural damage missense mutation identified in an ASV at the W198L region of the *erm(c)* gene as identified by Missense-3D [51]. The missense substitution detected in this ASV results in an amino acid change that expands the protein cavity volume by 85.32 Å^3. Location and 3D structure representation of the amino acid conformation is highlighted to show conformational changes in the protein structure of the *erm(C)* gene (A; highlighted in blue) due to a missense mutation (B; highlighted in red). An additional overlay of the original amino acid 3D structure and the altered amino acid structure is provided (C).

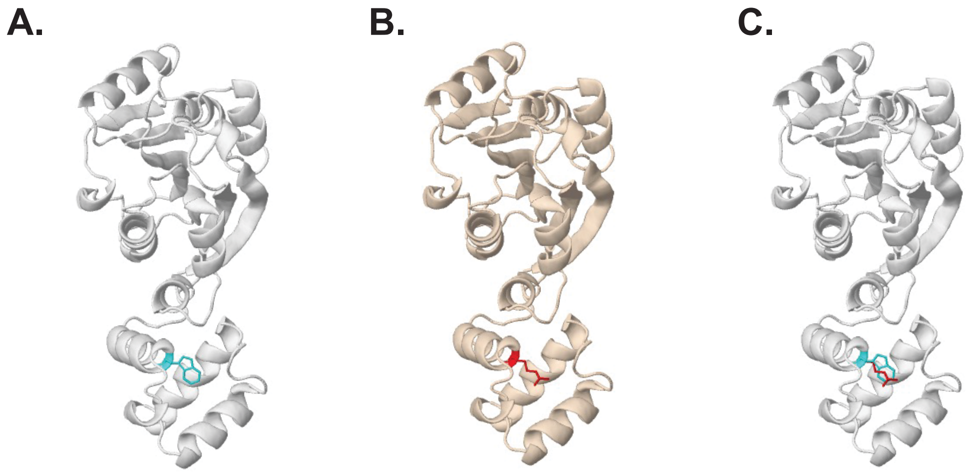

Supplementary Figure 7. Structural damage missense mutation identified in an ASV at the W198R region of the *erm(c)* gene as identified by Missense-3D [51]. The missense substitution detected in this ASV results in an amino acid change from a buried hydrophobic charged tryptophan residue (TRP, RSA 6.6%) to a buried uncharged hydrophilic arginine residue (ARG, RSA 6.4%). Location and 3D structure representation of the amino acid conformation is highlighted to show conformational changes in the protein structure of the *erm(C)* gene (A; highlighted in blue) due to a missense mutation (B; highlighted in red). An additional overlay of the original amino acid 3D structure and the altered amino acid structure is provided (C).
